# Supplementary material for: Slower maximal walking speed is associated with poorer global cognitive function among older adults residing in China
Source: PeerJ. 2022 Jul 26;10:e13809. doi: 10.7717/peerj.13809 (PMC9336608; doi:10.7717/peerj.13809)
Supplement: Data S2 — Used to interpret the meaning of the numbers in the raw data. [file peerj-10-13809-s002.docx]

**Raw data codebook**

The "0,1,2,3,4,5" in the "Education" list represent illiterate, primary, junior high, high school, and university respectively.

The "1,2,3,4,5,6" in the "Age Group" list represent the 60-64, 65-69, 70-74, 75-79, 80-84, and 85-89 age groups, respectively.
